# Characterization of *Actinobacillus pleuropneumoniae* isolates from pigs in Piedmont, Italy, by whole-genome sequencing: insights into antimicrobial resistance

**DOI:** 10.3389/fvets.2026.1861912

**Published:** 2026-08-11

**Authors:** Matteo Cuccato, Francesco Chiesa, Eugenio Mazzone, Sara Divari, Francesca Tiziana Cannizzo

**Affiliations:** Department of Veterinary Science, University of Turin, Grugliasco, Italy

**Keywords:** *Actinobacillus pleuropneumoniae*, AMR, Apx toxins, capsular polysaccharide, pangenome, porcine pleuropneumonia, WGS

## Abstract

**Background:**

*Actinobacillus pleuropneumoniae* (APP) remains a major respiratory pathogen in swine production worldwide. The limited cross-protection of available vaccines and the increasing occurrence of antimicrobial resistance (AMR) highlight the need for integrated phenotypic and genomic surveillance to support effective local control strategies.

**Methods:**

Twelve APP isolates previously collected in Piedmont (Italy) were investigated. Antimicrobial susceptibility was assessed by minimum inhibitory concentration (MIC) testing against a panel of antimicrobials commonly used in swine medicine. Whole-genome sequencing was performed using Illumina technology, followed by resistome profiling and comparative genomic analyses. Virulence-associated genes, including apx toxin genes and capsular polysaccharide loci, were also characterized.

**Results:**

Phenotypic testing revealed resistance to tetracycline, macrolides, *β*-lactams, tiamulin, and sulfamethoxazole/trimethoprim in a subset of strains. Genomic analysis identified tetracycline- and phenicol-resistance genes (*tet*, *flor*) in one isolate, while genes associated with macrolide and elfamycin resistance were widespread across the dataset. All isolates carried major apx toxin genes, with variable distribution of *apxII* and *apxIII* operons. A serovar 9/11 isolate showed a partial deletion within the capsular polysaccharide biosynthesis locus, confirmed by PCR and sequencing. Comparative genomics also suggested distinct genomic lineages among serovar 6 isolates.

**Conclusion:**

The combined phenotypic and genomic approach provides valuable insights for evidence-based antimicrobial use and provides additional genomic information on clinically relevant APP field isolates and supports future studies on AMR and genomic diversity.

## 1 Introduction

*Actinobacillus pleuropneumoniae* (APP) remains one of the most significant respiratory pathogens in the swine industry worldwide (1). This Gram-negative bacterium not only causes acute outbreaks with high morbidity and mortality, but also chronic forms with reduced weight gain, decreased feed efficiency, and substantial economic losses (2). Although the prevalence and distribution of individual APP serovars vary considerably among geographical regions, APP remains endemic in most pig-producing countries and is widely distributed in intensive swine production systems, contributing to recurrent clinical outbreaks and persistent subclinical infections (3). Beyond mortality, which may reach up to 10% during acute outbreaks, porcine pleuropneumonia is responsible for substantial economic losses due to reduced average daily gain, impaired feed conversion efficiency (up to 26%), increased treatment and vaccination costs, and overall reductions in farm profitability that may reach 34% in affected herds (4, 5). At farm level, effective control traditionally relies on vaccination plans and antimicrobial (AM) treatments (6). However, available commercial vaccines often provide only partial immunity and are serotype-specific, making it difficult to cover all circulating serotypes in a region (3). Based on the immunization mechanisms, two main types of vaccines have been developed to prevent APP infection: bacterins and subunit vaccines (7). However, both approaches have notable limitations: bacterins generally induce strong homologous immunity but limited heterologous cross-protection among the 19 APP serotypes (7, 8); while subunit vaccines can only reduce clinical signs and lung lesions but still fail to confer full protection (9). Additionally, vaccination plans may not adequately address less pathogenic or subclinical strains that can enter pig farms and remain undetected for the absence of overt lesions at both farm and slaughterhouse levels (10). In this context, the vaccine choice is mainly based on the main APP serotypes circulating in each country and the level of virulence and pathogenicity, confirming the fundamental link between serotyping and immunization protocols (11). Since commercial vaccines do not provide full protection against all APP serotypes, clinical outbreaks may still occur upon the introduction of new serotypes. In such cases, AM treatments remain the only available option to control the disease. Many AM molecules are active against APP (3); however, first choice AM therapies can vary among penicillins, tetracyclines, sulfonamides and amphenicols (12, 13). Other AM options, such as 3rd or 4th generation cephalosporins or fluoroquinolones, are restricted and permitted only when antimicrobial resistance (AMR) has been demonstrated in APP isolates (12, 13). Nevertheless, many studies reported the presence of resistant APP strains worldwide (14, 15). AMR is a growing concern in swine production, undermining treatment efficacy and potentially leading to higher mortality (2). In this scenario, where neither vaccination nor antimicrobial therapy alone ensures full protection against APP, defining the AMR profile of circulating strains becomes essential. Such data are critical both for guiding evidence-based therapeutic choices and for expanding the limited knowledge on AMR in clinically relevant swine pathogens (16). Another key aspect in APP pathogenicity is its virulence determinants. The virulence is multifactorial and influenced by host and environmental conditions, including co-infections with viral pathogens and specific pig lineages (4, 17). Nevertheless, the major determinants of pathogenicity are the pore-forming Apx toxins, which cause severe damage to host cells and tissues (2, 18). APP produces four Apx toxins (ApxI, ApxII, ApxIII, and ApxIV), each contributing differently to virulence. ApxI is both strongly hemolytic and cytotoxic, while ApxII shows moderate hemolytic and cytotoxic activity. ApxIII is highly cytotoxic but lacks hemolytic activity. Unlike the first three toxins, which are common to other *Actinobacillus* species, ApxIV is specific to APP, making it a marker of APP exposure (19). Considering this, a comprehensive understanding of both virulence traits and AMR profiles is crucial, since the coexistence of highly pathogenic and multidrug-resistant strains would represent a serious threat for animal health, farm productivity, and the sustainability of treatment strategies.

The main aim of this study was to provide an integrated phenotypic and whole-genome characterization of APP isolates previously recovered during our local epidemiological survey in Piedmont (20). Rather than estimating the epidemiology of APP in the region, the present study was designed to generate a detailed genomic and phenotypic description of representative clinical isolates recovered during the previous survey.

## 2 Materials and methods

### 2.1 APP isolation and DNA extraction

A total of 12 cryopreserved APP samples, previously isolated and described by Cuccato and colleagues (20), were inoculated on 5 mL of Brain Heart Infusion (BHI) broth supplemented with 0.01% NAD (8). The isolates included in the present study originated from different commercial pig farms distributed throughout the Piedmont region and were recovered during the regional survey previously cited. Serotypes of the investigated APP strains (Table 1) were previously characterized according to two multiplex PCRs as described in Stringer et al. (8).

**Table 1:** Serovar distribution of the 12 APP isolates.

ID	Serovar
ap 11,472	2
ap 2,686	6
ap 3,656	2
ap 4,658	2
ap 5,778	9/11
ap 6,262	2
ap 7,682	6
ap 8,657	2
ap 91,054	6
ap 10,683	6
ap 11,684	6
ap 12,574	2

After 24 h incubation at 37°C, tubes were centrifuged at 14,000 x g for 5 min and bacterial pellets were processed for DNA extraction by classical procedure involving detergent-mediated lysis, proteinase treatment, extraction and precipitation with isopropanol/ethanol (21). In brief, bacterial pellet was re-suspended with 500 μL of a rapid lysis buffer (200 mM NaCl, 100 mM Tris–HCl pH 8.0, 5 mM EDTA, 0.2% SDS) and 5 μL of proteinase K (20 mg/mL) and incubated overnight at 56 °C. Then the DNA was precipitated using isopropanol and was washed twice using 70 and 100% ethanol, respectively. Extracted DNA was diluted in 100 μL of nuclease-free water and analyzed using a Nanodrop spectrophotometer (Thermo Fisher Scientific, Waltham, MA, United States). DNA concentration was also determined using the QuantiFluor® dsDNA System (Promega, Madison, WI, United States) using the Quantus Fluorometer (Promega, United States). Samples exhibiting 260/280 and 260/230 ratios greater than 1.8 were considered of sufficient quality and selected for whole-genome sequencing (WGS).

### 2.2 Phenotypic AMR profiles determination of APP isolates

Isolates were processed and tested by an accredited external laboratory following standard procedures for minimum inhibitory concentration (MIC, μg/mL) on swine respiratory pathogens. Briefly, APP strains were revitalized and suspended by broth microdilution using the Sensititre semi-automated system (Thermo Fisher Scientific, United States). The AM panel included gamithromycin, tiamulin, tildipirosin, tilmicosin, tulathromycin, amoxicillin/clavulanic acid, ampicillin, ceftiofur, enrofloxacin, florfenicol, flumequine, kanamycin, spectinomycin, sulfamethoxazole/trimethoprim, and tetracycline. Results were interpreted according to the Clinical and Laboratory Standards Institute guidelines (22). Phenotypic AMR profiles of APP isolates were classified as susceptible, intermediate and resistant.

### 2.3 WGS and sequence data processing

WGS was conducted by an external company using Illumina HiSeq 2,500 platform. DNA libraries were prepared according to standard Illumina protocols, and sequencing was performed to produce paired-end reads of approximately 150 bp. The raw reads were provided in FASTQ format and subjected to quality control using FastQC and MultiQC. Raw sequencing reads were obtained in FASTQ format and subjected to preprocessing and AMR gene profiling using the AMR++ pipeline (23) in conjunction with the MegaRes database (24). The pipeline incorporates a series of steps designed to ensure accurate and reproducible downstream analysis. Initially, the reads underwent quality assessment and adapter removal. Low-quality bases and short reads were trimmed according to the standard thresholds implemented in the pipeline using Trimmomatic (25). These procedures ensured that only high-quality reads were retained for subsequent analyses.

### 2.4 Comparative genomic analysis

*De novo* genome assemblies were generated from the Illumina paired-end reads using Unicycler (v0.5.0) under default hybrid-assembly parameters (26). Genome assembly quality was assessed using QUAST (v5.2.0), which provided assembly statistics including total genome length, GC content, number of contigs, and N50 values (27). Genome completeness and contamination were assessed using CheckM (v1.2.5) with the lineage-specific workflow (lineage_wf) (28). The assembled genomes were imported into Anvi’o (v8) for visualization and comparative genomic analysis (29). A pangenomic workflow was generated using the “anvi-pan-genome” module with default parameters for gene clustering. Average Nucleotide Identity (ANI) was calculated between each isolate and reference genomes representing four major APP serovars: serovar 11 (strain 56,153, GenBank accession CP031863.1), serovar 9 (strain CVJ13261, CP031865.1), serovar 6 (strain Femo, MZ450073.1), and serovar 2 (strain S1536, CP031875.1). Pairwise ANI values and hierarchical clustering were used to assign each isolate to the most closely related reference serovar. Genomic relationships were visualized as interactive pangenomic maps in Anvi’o, allowing for the simultaneous exploration of gene presence/absence and sequence similarity patterns.

### 2.5 Phylogenetic analysis

To further investigate the genomic relationships among the isolates, a maximum-likelihood phylogenetic tree was reconstructed from concatenated single-copy core gene clusters identified during the anvi’o pangenome analysis. Core gene amino acid sequences were extracted using anvi-get-sequences-for-gene-clusters with filters retaining only single-copy genes present in all genomes, concatenated, and analyzed with IQ-TREE (v3.1.2). The best-fit substitution model was selected using ModelFinder, and branch support was estimated with 1,000 ultrafast bootstrap replicates.

### 2.6 Virulence factor profiling

A preliminary evaluation of publicly available virulence databases, including the Virulence Factor Database (VFDB), was performed to identify a suitable reference dataset for APP. However, no curated species-specific virulence gene dataset is currently available for APP. Therefore, a custom reference library was generated using publicly available nucleotide sequences of the best-characterized APP virulence determinants, namely the APX toxin-associated genes (30). These genes were selected because they represent the major virulence factors currently associated with APP pathogenicity and disease severity. Virulence-associated genes were investigated using the fromAssembly2feature pipeline, applying a curated list of APP virulence genes belonging to apxI, apxII, and apxIII groups. The presence, completeness, and sequence variants of these genes were determined through sequence alignment against the assembled genomes. The capsular polysaccharide biosynthesis region, which defines the APP serovar, was identified and extracted from each assembled genome using a custom Python script. The script located the region based on genome annotation boundaries, specifically the intergenic interval between two conserved flanking genes used as reference markers. Each extracted region was annotated using Prokka (v1.14.6), and the predicted open reading frames (ORFs) were compared among isolates and reference strains (31).

### 2.7 Resistome profiling

Following preprocessing, the high-quality reads were mapped to the MegaRes database, which provides a curated and hierarchically organized catalog of AMR genes. Gene-level alignments were performed using the Burrows-Wheeler Aligner integrated within AMR++, and AMR determinants were identified based on the default thresholds for sequence similarity and coverage (namely 80% and 20x) (31). The resulting alignments were used to generate a resistome profile for each dataset, providing both qualitative (presence/absence) and quantitative (relative abundance) information on AMR genes.

### 2.8 Sanger sequencing

Comparative sequence analysis revealed that the isolate assigned to the serovar 9/11 group carried a partial deletion within the capsular region, affecting several genes involved in polysaccharide biosynthesis. To verify this genomic alteration, specific primers were designed using Primer3 (32), and the target region was amplified by endpoint PCR followed by Sanger sequencing. A 291 bp segment was amplified using the primers APf (5′- TGAGCAAGCAGAAAGGAACA-3′) and APr (5′- ACCTGAAAACTATTCCAAGCGA-3′). The HotStarTaq Master mix (Qiagen, Hilde, DE) was used with 0.2 μM of each primer and 1 μl of DNA template in a final volume of 20 μL. The cycling conditions were 15 min at 95 °C, followed by 30 cycles of 95 °C for 30 s, 58.5 °C for 30 s, and 72 °C for 40 s. At the end of the reaction, an additional extension step at 72 °C for 7 min was applied. PCR product was visualized by electrophoresis, purified through MinElute PCR purification kit (Qiagen) and sequenced in both directions using Sanger method by an external sequencing provider.

## 3 Results

### 3.1 Phenotypic antimicrobial susceptibility profiles

Although 20 APP isolates were initially obtained in the previous study (20), only 12 could be successfully re-cultured after cryopreservation and yielded sufficient high-quality DNA for downstream WGS analysis. Phenotypic antimicrobial susceptibility testing by MIC revealed heterogeneous AMR profiles among the 12 APP isolates (Supplementary Table S1). Resistance to macrolides and related compounds was variably detected: resistance to tilmicosin and tulathromycin was observed in 2/12 isolates each, while all remaining isolates were classified as susceptible or intermediate according to CLSI breakpoints. Resistance to tiamulin was detected in 2/12 isolates. Among *β*-lactam antibiotics, resistance to amoxicillin/clavulanic acid and ampicillin was observed in 2/12 isolates for each compound, whereas all isolates were susceptible to ceftiofur. Resistance to kanamycin was identified in one isolate (1/12). Resistance to sulfamethoxazole/trimethoprim was detected in 2/12 isolates. Tetracycline resistance represented the most frequently observed phenotype, occurring in 3/12 isolates. No resistance was detected for enrofloxacin, flumequine, spectinomycin, florfenicol, gamithromycin, or tildipirosin.

### 3.2 Virulence factor profiles

Virulence factor profiling revealed variability in the distribution of APX toxin–encoding genes among the analyzed isolates. The *apxIB* and *apxID* genes were detected in all 12 isolates (Supplementary Table S2). The *apxIC* gene was identified in only one isolate. The *apxIIC* and *apxIIIA* genes were present in 11/12 isolates. Genes encoding the APX III secretion apparatus components showed greater variability, with *apxIIIB* detected in 6/12 isolates and *apxIIID* in 5/12 isolates.

### 3.3 Genotypic AMR profiling results

WGS–based resistome profiling identified a limited but consistent set of AMR genes among the APP isolates (Supplementary Table S3). Genes associated with resistance to tetracyclines and phenicols, including *tetB*, *tetM*, and *flor*, were detected in only one isolate (serovar 9/11). Genes associated with macrolide resistance were more widely distributed. A *16S rRNA* methylation–associated resistance gene was identified in 6/12 isolates. In contrast, the mls23S gene, conferring resistance to macrolides, and the *tufAB* gene, associated with elfamycin resistance, were detected in all analyzed isolates. Both determinants are classified by MegaRes as “requires SNP,” indicating that their presence alone is not sufficient to predict a resistant phenotype, as resistance depends on the occurrence of specific resistance-conferring point mutations.

### 3.4 Comparative genome analysis and Sanger sequencing results

Genome assemblies showed consistently high quality, with genome sizes ranging from 2.22 to 2.29 Mb, GC contents of approximately 41%, N50 values between 112 and 171 kb, and 24–37 contigs per assembly. CheckM assigned all isolates to the family *Pasteurellaceae* (UID4932), with estimated completeness values ranging from 99.66 to 99.89% and no detectable contamination (Supplementary Table S4).

**Figure 1:**
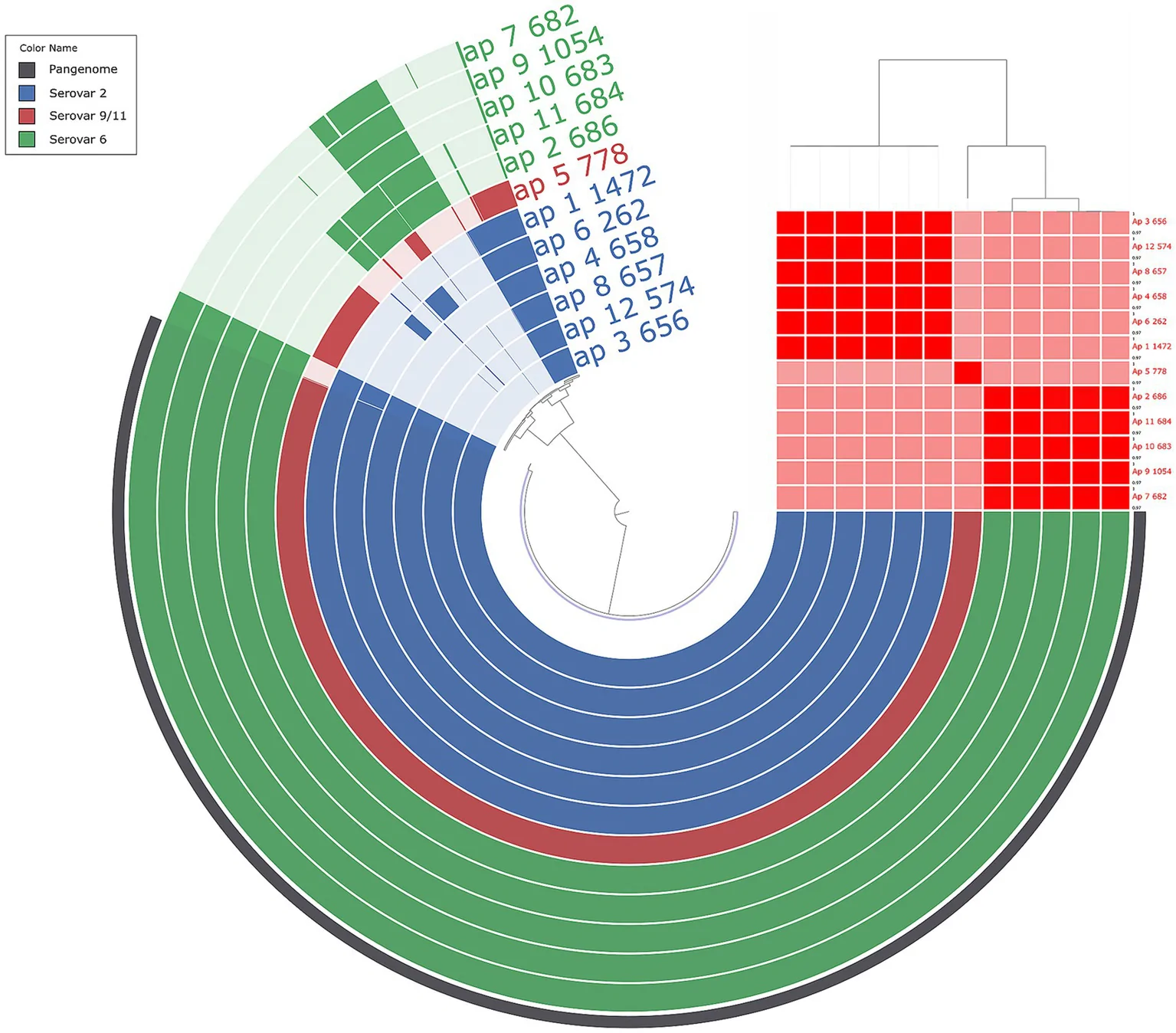
Pangenome-based comparative genomic analysis of APP isolates. The circular representation shows gene presence/absence across genomes, with isolates color-coded according to serovar (serovar 2 = blue; serovar 9–11 = red; serovar 6 = green). The hierarchical clustering and heatmap illustrate the genetic relationships and variability in gene content among isolates.

Comparative genomic analysis based on *de novo* assemblies and Average Nucleotide Identity (ANI) assigned the isolates to their closest reference serovars. Pangenome analysis highlighted distinct clustering patterns among isolates, with strains grouping according to serovar and showing variability in gene presence/absence profiles, supporting the existence of genetically distinct lineages within the same serovar (Figure 1).

**Figure 2:**
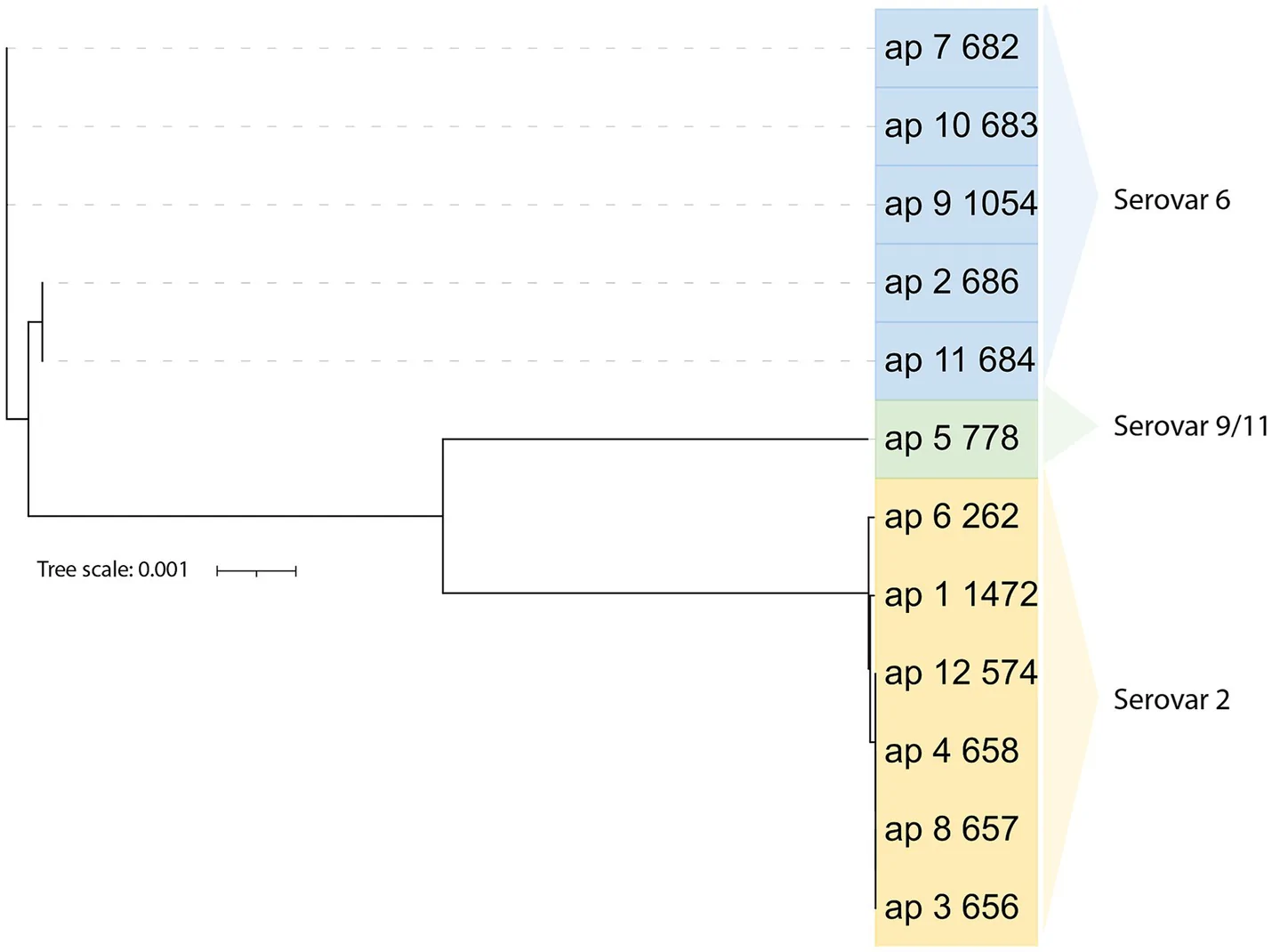
Maximum-likelihood phylogenetic tree of the 12 APP isolates based on concatenated amino acid sequences of single-copy core genes. Isolates are color-coded according to serovar (serovar 2 = blue, serovar 6 = yellow, serovar 9/11 = green).

A maximum-likelihood phylogenetic tree based on concatenated single-copy core genes was reconstructed to further investigate genomic relationships among isolates (Figure 2). The resulting topology was consistent with the pangenome analysis, clustering isolates according to serovar while also supporting the presence of distinct genomic lineages within serovar 6.

**Figure 3:**
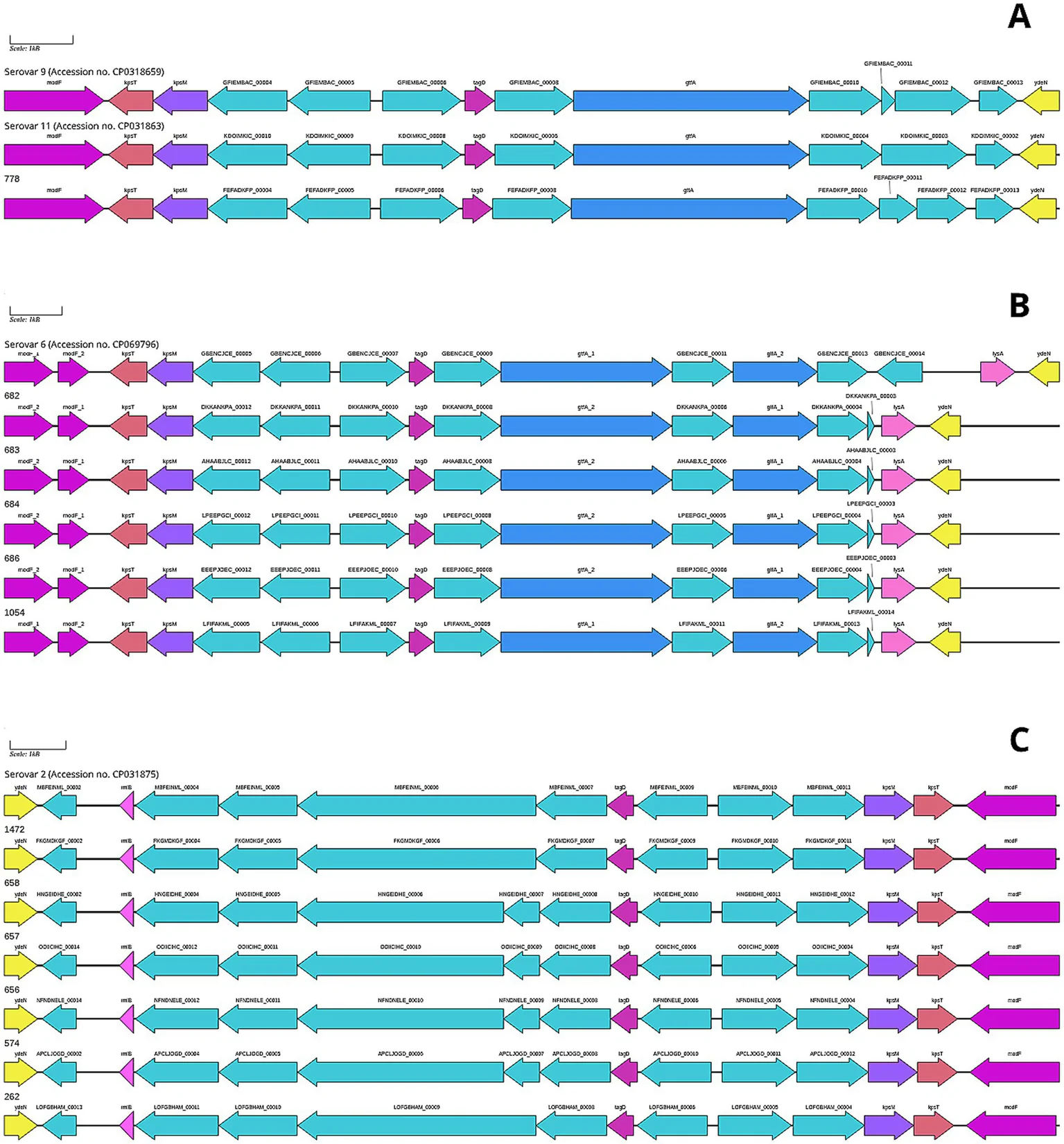
Comparative genomic analysis on cps locus of APP isolates. Visualization of the cps locus in the serovar 9/11 isolate showing a partial deletion confirmed by Sanger sequencing **(A)**. Pangenome-based analysis of serovar 6 isolates highlighting variability in gene presence/absence and suggesting distinct genomic lineages **(B)**. Comparative representation of gene content in serovar 2 isolates, showing a more conserved genomic profile **(C)**. Reference APP strains were included for comparison of genomic loci (45).

One isolate belonging to the serovar 9/11 group exhibited a partial deletion within the capsular polysaccharide biosynthesis (*cps*) locus (Figure 3A). This deletion was confirmed by targeted PCR amplification and Sanger sequencing, which validated the absence of a 291 bp region within the cps locus. Isolates classified as serovar 6 showed genomic differences in the anvi’o-based pangenomic analysis, including variation in gene presence/absence patterns, suggesting distinct genomic backgrounds among these strains (Figure 3B). Serovar 2 APP strains showed a more conserved genomic profile on cps locus (Figure 3C).

## 4 Discussion

In the latest years, increasing attention has been directed toward APP and porcine pleuropneumonia in the global pig industry (2). Serotyping is now recognized as a key practice for monitoring APP circulation (8), particularly given the coexistence of 19 distinct serovars and the consequent implications for the choice of an appropriate vaccination plan. In a previous study conducted in North-West Italy, we described for the first time the prevalence of circulating APP serovars in this Region (20). Notably, the attention of that investigation was focused on the farm-level rather than the slaughterhouse-based surveillance, which is more commonly the epidemiological checkpoint for this disease (33, 34). This approach revealed a different epidemiological scenario, characterized by a different prevalence of APP infection, distinct circulating serovars and more severe lesions compared to those typically reported at the abattoir level (20). In this context, the characterization of AMR profiles of APP strains, previously isolated in Piedmont, can provide additional insights useful for the pathogen management. The findings of the present study should be interpreted considering the descriptive nature of the study design. Unlike our previous work (20), which investigated the circulation and serovar distribution of APP in pigs submitted for necropsy after natural death on commercial farms, the objective of the present study was not to estimate the regional epidemiology of APP. Instead, this work was designed as a genomic follow-up investigation aimed at providing an integrated phenotypic and whole-genome characterization of the available APP isolates recovered during that survey. In the previous investigation, 107 pigs originating from 40 independent commercial fattening farms distributed across the Piedmont region were examined, and 20 APP isolates were successfully recovered (20). Of these, only 12 isolates could subsequently be successfully re-cultured after cryopreservation and yielded sufficient high-quality DNA for whole-genome sequencing, reflecting the critical growth characteristics of APP and the stringent DNA quality requirements necessary for downstream genomic analyses. Therefore, the present findings should be considered descriptive and hypothesis-generating, providing additional insight into the genomic characteristics, virulence-associated features, and antimicrobial resistance determinants of clinically relevant APP field isolates (35–37).

Overall, the phenotypic AMR profile observed in the present study highlighted the occurrence of resistance to several antimicrobial classes, although with variable frequency. Among the tested compounds, tetracycline showed the highest frequency of resistance, followed by tiamulin, tilmicosin, tulathromycin, amoxicillin/clavulanic acid, ampicillin, and trimethoprim/sulfamethoxazole, whereas kanamycin resistance was detected only in one strain. These results are consistent with previous studies identifying tetracyclines as one of the antimicrobial classes most frequently associated with reduced susceptibility in APP (14, 37). In a recent Italian study on isolates collected from pig farms between 2015 and 2022, tetracycline resistance was the most common phenotype (53%), followed by ampicillin (33%) and trimethoprim/sulfamethoxazole (23%), with multidrug AMR commonly observed (46). Compared with those findings, the isolates analyzed here showed a lower frequency of resistance for these compounds, suggesting a less severe AMR scenario in the investigated population. While AMR represents a key aspect of APP characterization, the pathogenic potential of circulating strains is also strongly influenced by their virulence-associated gene repertoire. Virulence factor profiling revealed a heterogeneous distribution of APX toxin–associated genes among the analyzed isolates. The consistent detection of *apxIB* and *apxID* in all strains is in line with their essential role in the secretion and activation of RTX toxins in APP strains (46). These genes encode components of the secretion system and are typically conserved across strains, regardless of serovar (18). In contrast, the distribution of toxin structural genes showed greater variability. The *apxIIC* and *apxIIIA* genes were detected in the majority of isolates, whereas *apxIIIB* and *apxIIID* were present in approximately half of the strains. In particular, isolates belonging to serovar 6 tended to harbor a broader repertoire of APX-associated genes, including a higher frequency of *apxIIIB* and *apxIIID*, whereas serovar 2 isolates displayed a comparatively reduced and more variable toxin gene profile. This variability is consistent with previous studies reporting that the composition of APX toxin operons differs among serovars and contributes to differences in virulence profiles (8, 46). The presence of multiple APX toxin genes in most isolates supports their central role in APP pathogenicity, particularly in lung tissue damage and disease severity (39–41). However, the heterogeneous distribution observed in this study suggests that not all strains share the same virulence potential. While the detection of these genes provides useful information on the genetic repertoire of the isolates, their actual contribution to pathogenicity may depend on gene expression levels and host-related factors (17). These findings further support the concept that serovar classification alone may not fully capture the diversity of virulence-associated gene profiles among APP strains. In addition, the present analysis focused on APX-associated genes because these represent the best-characterized virulence determinants of APP and no comprehensive, curated species-specific virulence database is currently available to support broader virulome analyses. Future studies may expand this approach as more comprehensive genomic resources become available for this species.

WGS–based resistome analysis revealed a relatively conserved distribution of AMR genes among the analyzed isolates, despite the variability observed at the phenotypic level. In particular, genes associated with resistance to tetracyclines and phenicols (*tetB*, *tetM*, and *flor*) were detected in one isolate (serovar 9/11), whereas tetracycline resistance was the most frequently observed phenotype. This apparent discrepancy between genotype and phenotype has been previously reported in APP and other bacterial species and may reflect differences in gene expression, regulatory mechanisms, or the presence of alternative AMR determinants not captured by current databases (42). In contrast, genes associated with macrolide resistance were more widely distributed. The detection of *mls23S* and *tufAB* genes in all isolates, together with the presence of a 16S rRNA methylation–associated gene in half of the strains, suggests a conserved genomic background potentially related to macrolide and elfamycin resistance. Importantly, the mls23S and tufAB determinants identified in the present study are classified in the MegaRes database as “requires SNP” genes. Consequently, the presence of these loci alone is insufficient to predict phenotypic resistance, since the standard AMR++ workflow does not evaluate whether the associated sequence variants required for resistance are present. This aspect may partially explain the apparent discordance observed between the WGS-derived resistome and the phenotypic susceptibility profiles. However, the incomplete concordance with phenotypic susceptibility results indicates that the presence of these genes alone may not be sufficient to confer resistance under standard testing conditions, as also reported in APP using combined phenotypic and WGS approaches, where concordance between genotype and phenotype may vary depending on gene expression, genomic context, and the presence of mobile genetic elements (30, 43).

Comparative genomic analysis further highlighted the genetic diversity among the analyzed isolates. The identification of a partial deletion within the cps locus in the serovar 9/11 isolate, confirmed by Sanger sequencing, represents a noteworthy finding. Given the central role of the capsular polysaccharide in serovar determination and immune evasion, structural alterations in this region may have implications for both strain classification and host–pathogen interactions (30). Notably, the serovar 9/11 isolate was the strain carrying the highest number of AMR genes and, at the same time, exhibited a partial deletion within the *cps* locus. Although no direct association between capsular locus alterations and AMR gene accumulation can be inferred from the present data, this co-occurrence suggests that this isolate may represent a particular genomic profile. This interpretation is consistent with previous studies showing that the *cps* locus is a highly variable region shaped by gene acquisition and deletion, and that AMR determinants in APP are frequently associated with plasmids and other mobile genetic elements (30, 43). To further clarify, all genome assemblies were also analyzed using the PlasmidFinder pipeline (data not shown). However, no plasmid-associated sequences were identified in the analyzed isolates. Nevertheless, this finding does not completely exclude the presence of plasmids, as short-read sequencing and current plasmid databases may fail to reconstruct or identify all plasmid structures. Complementary approaches, including long-read sequencing and transcriptomic analyses, will be valuable in future studies to further investigate these mechanisms.

In addition, recent evidence indicates that serovar 9/11 isolates may display atypical *cps* patterns due to deletions affecting capsular genes, further supporting the biological relevance of the structural alteration observed in the present study (44). Beyond AMR, comparative genomic analysis also revealed structural variations affecting key regions involved in strain classification and host interaction. Additionally, isolates assigned to serovar 6 displayed variability in gene content based on pangenomic analysis, suggesting the presence of distinct genomic lineages within the same serovar. The core-genome phylogenetic analysis further supported the comparative genomic findings by confirming the clustering of isolates according to their serovar assignment. In particular, serovar 2 isolates showed a highly homogeneous phylogenetic profile, whereas serovar 6 isolates displayed greater genomic variability, forming two closely related but distinguishable phylogenetic subclusters. These observations are consistent with the pangenome analysis and suggest that isolates sharing the same serovar may still exhibit measurable genomic heterogeneity. This observation is consistent with previous reports indicating that isolates sharing the same serovar classification may exhibit considerable genomic heterogeneity (43, 46). These findings further support the notion that serovar classification alone may not fully capture the genomic variability among APP isolates and that whole-genome sequencing provides additional resolution for their characterization. Nevertheless, the potential effects of this genomic variation on capsule biosynthesis, host–pathogen interactions, or bacterial virulence remain to be experimentally validated through dedicated *in vitro* and/or *in vivo* functional studies.

## 5 Conclusion

In conclusion, this study provides an integrated phenotypic and genomic characterization of APP isolates, recovered during a previous regional investigation in Piedmont (20), expanding the genomic characterization of clinically relevant APP field isolates. The combined phenotypic and genomic approach profile revealed the occurrence of antimicrobial resistance determinants, characterized the distribution of major APX-associated virulence genes, and revealed genomic variability among isolates belonging to the same serovar., Comparative genomic analysis further highlighted this diversity by identifying a partial deletion within the *cps* locus of a serovar 9/11 isolate and distinct genomic lineages among serovar 6 isolates. Overall, this study highlights the value of integrating antimicrobial susceptibility testing with genomic approaches to improve the characterization of AMR determinants, virulence-associated features, and genomic variability in APP. Given the descriptive nature of the study and the limited number of available isolates, these findings should be interpreted as a genomic characterization of clinical field isolates and provide a foundation for the future integrated strategies in the management of swine pleuropneumonia.

## Data Availability

Genome assemblies data was deposited at NCBI under BioProject accession number PRJNA1453976. All the remaining data presented in this study are available directly in the article or in the Supplementary material. Other relevant information can be obtained from the corresponding author upon request.
