# Supplementary material for: Characterization of Actinobacillus pleuropneumoniae isolates from pigs in Piedmont, Italy, by whole-genome sequencing: insights into antimicrobial resistance
Source: Front Vet Sci. 2026 Aug 11;13:1861912. doi: 10.3389/fvets.2026.1861912 (PMC13503222; doi:10.3389/fvets.2026.1861912)
Supplement: Supplementary file 1 [file Table_4.DOCX]

| **Isolate** | **Genome size (bp)** | **Contigs** | **GC (%)** | **N50 (bp)** | **Completeness (%)** | **Contamination (%)** |
| --- | --- | --- | --- | --- | --- | --- |
| ap 1 1472 | 2,227,712 | 29 | 41.13 | 146,283 | 99.66 | 0.00 |
| ap 2 686 | 2,283,026 | 28 | 41.05 | 139,726 | 99.89 | 0.00 |
| ap 3 656 | 2,220,250 | 29 | 41.12 | 134,767 | 99.66 | 0.00 |
| ap 4 658 | 2,220,736 | 29 | 41.12 | 134,767 | 99.66 | 0.00 |
| ap 5 778 | 2,289,510 | 37 | 41.17 | 112,763 | 99.89 | 0.00 |
| ap 6 262 | 2,273,986 | 31 | 41.12 | 146,219 | 99.89 | 0.00 |
| ap 7 682 | 2,223,910 | 28 | 41.07 | 139,634 | 99.66 | 0.00 |
| ap 8 657 | 2,224,992 | 25 | 41.10 | 171,099 | 99.66 | 0.00 |
| ap 9 1054 | 2,227,071 | 27 | 41.05 | 139,634 | 99.66 | 0.00 |
| ap 10 683 | 2,229,340 | 24 | 41.05 | 167,715 | 99.66 | 0.00 |
| ap 11 684 | 2,279,594 | 30 | 41.05 | 121,679 | 99.89 | 0.00 |
| ap 12 574 | 2,225,081 | 29 | 41.11 | 134,767 | 99.66 | 0.00 |

**Supplementary Table S4.** Assembly quality metrics of the 12 APP genomes included in this study. Genome size, number of contigs, GC content and N50 were obtained using QUAST (v5.2.0). Genome completeness and contamination were estimated using CheckM (v1.2.5).
